# Physiomorphology of Soybean as Affected by Drought Stress and Nitrogen Application

**DOI:** 10.1155/2020/6093836

**Published:** 2020-04-10

**Authors:** Oqba Basal, András Szabó

**Affiliations:** Department of Crop Production and Applied Ecology, University of Debrecen, Debrecen, 4032/Böszörményi Road 138/A, Hungary

## Abstract

Drought periods are predicted to increase in the future, putting the production of sensitive crops under serious hazards. Soybean, as a legume, is capable of partly achieving its nitrogen demands through the N_2_-fixation process; however, this process is inhibited by drought stress conditions. Moreover, N_2_-fixation might not fulfill the total N demand for soybean plants, so supplemental N-fertilizer doses might be crucial. A 3-year experiment was carried out in Debrecen, Hungary, to investigate the effects of inoculation and N-fertilizer application on the physiomorphology of soybean (cv. Boglár) under both drought stress and irrigated conditions. Results showed that, regardless of inoculation, drought negatively affected plant height, LAI, SPAD, and, to a smaller extent, NDVI. On average, increasing N-fertilizer enhanced these traits accordingly. Inoculation, on the other hand, resulted in taller plants and higher LAI values, but lower SPAD values. It could be concluded that soybean's physiomorphology is negatively influenced by drought stress and that N-fertilizer application can enhance it whether soybean plants suffer from drought stress conditions or not.

## 1. Introduction

Legumes are known to improve soil fertility by symbiotic N_2_-fixation [1], and soybean (*Glycine max* (L.) Merrill) is one of the most important food legumes with its high protein and oil concentrations in the seeds [2]. However, soybean plants have high nutrient demand, especially nitrogen (N) [3]. The two main sources of nitrogen for soybean plants are biologically fixed N_2_ and mineral N-fertilizer [4]. One benefit of fixed N_2_ is that plants immediately use it, with no potential losses due to any environmental factor. Another point is that commercial inocula are much cheaper than chemical N- fertilizer [2]. Although some researchers reported that inoculated soybean does not need N-fertilizer application [5, 6], yet others reported otherwise [7–9] as fixed N_2_ was reported to provide soybean plants, on average, with 50–60% of required N [4]. Moreover, the inoculation process can enhance the plant's resistance to abiotic stresses [10]. It was previously reported that high rates of N-fertilizer inhibit the N_2_-fixation process, whereas a relatively low dose at the early stages of soybean development can be beneficial as the N_2_-fixation process will not be initiated by that time yet [2, 8]. Soybean is susceptible to drought [11], and drought intensities are predicted to increase [12], putting its production under serious challenges. Moreover, drought can negatively affect N_2_-fixation [13]. Besides this, many other physiomorphological traits can be influenced by drought, such as chlorophyll production [14], plant height [15, 16], and leaf area index [17]. Understanding crop's responses to drought stress can lead to better irrigation water exploitation and, consequently, better yields even under drought stress conditions [18], so this study aimed to screen the effects of drought stress and nitrogen application through two different resources, inoculation and mineral N-fertilizer, on the physiomorphology of soybean (cv. Boglár) in Debrecen, Hungary.

## 2. Materials and Methods

Soybean (cv. Boglár, Bonefarm, Hungary) was sown in a field experiment in the experimental station of the University of Debrecen (Látókép) (N. latitude 47^o^ 33′, E. longitude 21^o^ 27′) during 2017, 2018, and 2019 growing seasons. The soil type of the site is calcareous chernozem.

**Figure 1:**
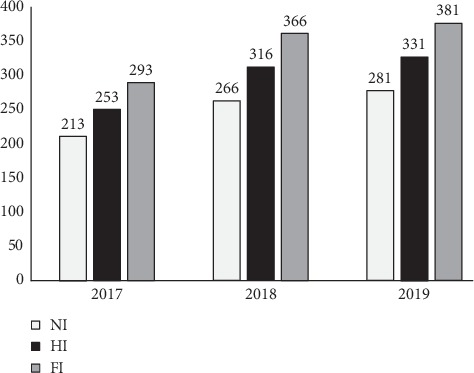
Irrigation amounts during the vegetative period of soybean (cv. Boglár) in 2017, 2018, and 2019 in Debrecen, Hungary. NI: nonirrigated; HI: half-irrigated; FI: fully irrigated.

The experimental design was a split-split-plot design. Three irrigation regimes, nonirrigated, half-irrigated, and fully irrigated (NI, HI, and FI, respectively), represented the main plots. Two inoculation treatments inoculated with *Bradyrhizobium japonicum* inoculant and noninoculated represented the subplots. Three N-fertilizer (NH_4_NO_3_) rates, 0, 35, and 105 kg ha^−1^ N (0N, 35N, and 105N, respectively), represented the sub-subplots with 4 replications each. NI treatment received only precipitation as water irrigation amount, whereas HI treatment received, in addition to precipitation, a total of 40 mm of irrigation water in 2017 and 50 mm in 2018 and 2019. FI treatment, on the other hand, received, in addition to precipitation, a total of 80 mm of irrigation water in 2017 and 100 mm in 2018 and 2019 (Figure 1).

Final plot number was 72 (3 irrigation regimes ^*∗*^ 2 inoculation treatments ^*∗*^ 3 fertilization rates ^*∗*^ 4 replications). The plot area was 49.68 m^2^ with 12 rows in each plot.

LAI values were recorded using the SS1 SunScan canopy analysis system (Delta- *T* Devices, UK). Relative chlorophyll content (in the form of SPAD) was measured using SPAD-502Plus (Konica Minolta, Japan). NDVI values were recorded using Trimble GreenSeeker Handheld (AS Communications Ltd., UK). Ten randomly selected plants from the middle rows of each plot were used for the mentioned traits. All traits were measured at four different stages of soybean's life cycle [19]: fourth node (V4), full bloom (R2), full pod (R4), and full seed (R6). Plant height was measured at the R6 stage using a standard ruler on 10 randomly selected plants from the middle rows of each plot.

SPSS software was run to analyze and compare the means and to indicate the effect size, followed by Tukey's post hoc test to indicate the statistically different means (IBM SPSS ver.26, US software).

## 3. Results

### 3.1. Relative Chlorophyll Content (SPAD)

In inoculated plants at all studied stages, increased SPAD values could be recorded with increasing fertilization rates, with the high fertilization rate being significantly higher at late reproductive stages (R4 and R6) compared to 0N counterpart. On average, the SPAD value was 3.5 and 6.4% in 35N and 105N treatments, respectively, compared to 0N treatment (Table 1). A significant correlation between fertilization and SPAD trait at all stages was estimated (Table 2). A very similar conclusion was recorded in noninoculated plants, and the enhancement rate was 2.6 and 6.6% for 35N and 105N treatments, respectively, compared to 0N treatment (Table 1). The correlation coefficient with fertilization was positive and significant at all stages except for the R2 stage (Table 2).

Drought had a vulnerable and insignificant effect on SPAD values at the studied stages in inoculated plants but had a significant negative effect at R6 stage, where 7.7 and 11.8% reduction in SPAD value was recorded compared to half- and fully irrigated treatments, respectively. On average, irrigation increased SPAD values by 1.0 and 2.9% under half- and fully irrigated regimes, respectively, compared to the nonirrigated counterpart (Table 3). Only at the R6 stage was the correlation between irrigation and SPAD significant (Table 4). In noninoculated plants also, drought decreased SPAD value by 5.4 and 10.8% compared to half- and fully drought regimes, respectively (Table 3). A similar conclusion was recorded regarding correlation (Table 4).

Interestingly, noninoculated plants had higher SPAD values than inoculated counterparts in all fertilization treatments and under all irrigation regimes (Tables 1 and 3).

### 3.2. Normalized Difference Vegetation Index (NDVI)

Except for a slight, insignificant decrease in 105N compared to 35N counterpart, increased fertilization rate in inoculated plants was accompanied by increased NDVI values, with 105 N treatment being significantly higher than 0N treatment at V4 stage and significantly higher than both 0N and 35N treatments at R2 stage. Averaged over all stages, 1.3 and 2.2% higher NDVI values were recorded in 35N and 105N treatments, respectively, compared to 0N counterpart. In all fertilization treatments, a rapid increase in NDVI was recorded between V4 and R2 stages, followed by gradual reduction through later stages (Table 5). The correlation coefficient was highly significant at both V4 and R2 stages but started decreasing after to become slightly negative at the R6 stage (Table 2). Noninoculated plants responded positively to fertilization; however, no significance was recorded. A similar trend was recorded among stages for noninoculated plants (Table 5), and the correlation coefficient was insignificantly positive throughout all stages (Table 2).

In general, irrigation enhanced this trait in inoculated plants (except at the R2 stage, where also both irrigation regimes had higher NDVI value than the nonirrigated counterpart, but the half-irrigated regime had higher NDVI than did fully irrigated regime). Moreover, drought significantly reduced (by 5.3% compared to fully irrigated counterpart) NDVI value at the R6 stage. On average, drought reduced NDVI value by 1.5 and 2.0% compared to half- and fully irrigated regimes, respectively. The effect of irrigation on NDVI values through stages was similar to that of fertilization (Table 6). The correlation with irrigation was positive at all stages except for the R2 stage (Table 4). Irrigation's effect on noninoculated plants was more measurable at late reproductive stages (R4 and R6), but the only half-irrigated regime, on average, resulted in better NDVI than the drought-stressed counterpart. NDVI values reached their maximum at the R2 stage under both non- and half-irrigated regimes, whereas they reached the maximum at the R4 stage under the fully irrigated regime, but without reaching the maximum value of the other two regimes (Table 6). Correlation with irrigation was negative at both V4 and R2 stages but positive later at R4 and R6 stages (Table 4).

A very close average value of NDVI was recorded for both inoculated and noninoculated plants (Tables 5 and 6).

### 3.3. Leaf Area Index (LAI)

Enhanced LAI values could be recorded at all stages with increasing fertilization rate in both inoculated and noninoculated plants, with the high rate (105N treatment) having significantly higher values at both V4 and R2 stages and an average 18.8 and 14% higher LAI values compared to 0N and 35N treatments, respectively, in inoculated plants, and 14.9 and 8.0% in noninoculated plants. Regardless of inoculation, gradual increases in LAI values through plants' development were recorded, with a peak at the R4 stage in all fertilization treatments. A significant correlation at all studied stages, except for the late R6 stage, was estimated, regardless of inoculation.

In inoculated plants, the half-irrigated regime did not result in better LAI values at both V4 and R2 stages, but did at later stages. The fully irrigated regime, on the other hand, had higher LAI values at all stages compared to both other regimes. Irrigation increased LAI by 8.3 and 14.9% under half- and fully irrigated regimes, respectively, compared to the nonirrigated counterpart. A similar conclusion could be recorded in noninoculated plants at all stages except for the V4 stage, where the fully irrigated regime, in addition to the half-irrigated regime, could not enhance LAI. In this trait as well, irrigation followed a similar trend to fertilization effect throughout plants' development, regardless of inoculation (Table 7). The correlation coefficient gradually increased through stages to reach a highly significant peak at the R4 stage, followed by a reduction at the R6 stage that, however, kept it significant in inoculated plants, but not in noninoculated counterparts (Table 4).

Inoculated plants were, on average, 4% higher in LAI compared to noninoculated counterparts, but the difference was insignificant (Tables 7 and 8).

### 3.4. Plant Height

Both irrigation and fertilization, but not their interaction, had a highly significant effect on the plant height of inoculated plants, whereas both treatments, in addition to their interaction, had no significant effect on noninoculated plants. The correlation coefficient was positive, yet not significant, with both treatments, regardless of inoculation treatment.

In inoculated plants, both half- and fully irrigated regimes resulted in significantly taller plants compared to the nonirrigated counterpart, regardless of fertilization treatment. Compared to half-irrigated, however, the fully irrigated regime could enhance this trait only in 0N treatment, resulting in a similar enhancement average of 7.5% as compared to the nonirrigated regime. 46.0% of differences in plant height resulted from the different irrigation regimes. In noninoculated plants, similar enhancement, as a result of irrigation application, was recorded; however, no significant differences were recorded. Moreover, the half-irrigated regime resulted in taller plants than did the fully irrigated regime, regardless of fertilization treatment (Table 9).

Although not statistically significant, measurable enhancements in this trait were accompanied by increasing fertilization rate in inoculated plants. On average, 4.1 and 7.3% taller plants resulted from 35N to 105N treatments, respectively, as compared to 0N treatment. Fertilization was responsible for 38.7% of differences in plant height. Similar enhancements by fertilization treatments were recorded in noninoculated plants (Table 9).

Inoculation had no significant effect on this trait; however, inoculated plants were, on average, 1.8% taller than noninoculated plants (Table 9).

## 4. Discussion

Our results showed that half-irrigated regime resulted in slightly taller plants compared to the fully irrigated counterpart (except for inoculated plants in 0N treatment); however, drought stress (nonirrigated regime) decreased this trait, regardless of inoculation or fertilization, with the decrease being significant in inoculated plants. Iqbal et al. [20] concluded that decreasing available water at the R4 stage from 100 to 50% FC slightly increased plant height in soybean; however, further reduction to 20% FC resulted in shorter plants compared to both 10 and 50% FC. Sepanlo et al. [21] also reported that soybean plants had 29.6% shorter plants under drought stress imposed at the flowering stage. We also found that inoculated plants were, on average, taller than noninoculated counterparts and that fertilization, regardless of inoculation, increased the plant height. Abera et al. [22] compared soybean plants using 7 rhizobia isolates and a noninoculated control in an experiment conducted in 2 different sites. The authors reported that the plant height of all inoculated treatments was higher than noninoculated control at both experimental sites. A similar conclusion was also reported by Bekere and Hailemaria [23]. Significant increases in soybean plant height (by 21.1 and 23.7%) as a result of inoculation were reported by Adeyemi et al. [24] in pot and field experiments, respectively. Our results showed that plant height was enhanced by fertilization, regardless of inoculation. Virk et al. [25] reported that soybean plant height was insignificantly enhanced by N application. 30.4% significant reduction in plant height as a result of N deficiency was reported [26].

Drought reduced the average SPAD values in both inoculated and noninoculated plants. Fixed N_2_ decreases under drought stress, resulting in decreased N content in the leaves which, in part, leads to decreased photosynthetic capacity [27–29]. Drought stress reduced the SPAD value by 11% [30]. Total chlorophyll (chl_a+b_) decreased by 42.5% under drought stress conditions imposed at the flowering stage, whereas the reduction ratio was 15.7% when soybean plants suffered from drought stress at the pod filling stage [21]. Cerezini et al. [31] reported that chlorophyll content was higher in noninoculated plants than that in inoculated counterparts when soybean did not suffer from drought stress, which supports our findings. We found that, regardless of inoculation, fertilization resulted in better SPAD values. de Almeida et al. [26] concluded that N deficiency significantly reduced the relative chlorophyll content in soybean plants by 84.4%. Increasing the N rate resulted in better SPAD values at different stages in soybean [32]. A similar conclusion was reported by Kolvanagh et al. [33].

We found that noninoculated plants had higher NDVI under drought stress conditions. Similarly, Cerezini et al. [31] reported that NDVI decreased by 5.4% in inoculated plants compared to noninoculated counterparts under drought stress conditions. Drought resulted in relatively lower NDVI values compared to irrigated counterparts. Fertilization, on the other hand, enhanced NDVI in inoculated plants only. Camoglu et al. [34] reported that drought reduced NDVI on pepper plants. Saleem et al. [35] reported enhanced NDVI as a result of N application on wheat plants, and a similar conclusion was reported on maize [36].

Decreased LAI values were recorded under drought stress, regardless of inoculation. Atti et al. [30] also concluded that two drought stress severities, W1 and W2 (corresponding to 25 and 50% of crop evapotranspiration (ET_c_)), reduced leaf area by 74.5 and 52.7%, respectively. Gavili et al. [37] reported that moderate and severe drought (corresponding to 70 and 55% FC, respectively) significantly decreased plant leaf area at all three studied stages. Severe drought stress imposed at the R4 stage resulted in 61.4% less leaf area in soybean [18]. Pagter et al. [38] explained the decreased LAI under drought stress conditions to be the result of less newly produced leaves with a smaller size and a higher falling rate. Regardless of inoculation, increasing the fertilization rate was accompanied by increased LAI. The application of N-fertilizer significantly increased LAI in soybean [25]. Caliskan et al. [8] concluded that soybean LAI linearly increased with increased N rates, whereas de Almeida et al. [26] found that the deficiency of N in soybean plants significantly decreased LAI by 87.5%.

## 5. Conclusions

Both irrigation and fertilization had a highly significant effect on the plant height of inoculated plants, but not on noninoculated plants. Drought decreased plant height, regardless of inoculation, but its effect was more recordable on inoculated plants. Fertilization enhanced this trait as well, and inoculated plants were insignificantly taller than noninoculated counterparts. The correlation coefficient was positive with both irrigation and fertilization, regardless of inoculation treatment.

Physiological traits were also affected by both fertilization and irrigation; LAI was more affected compared to SPAD and NDVI. Increasing fertilization rates and irrigation water amounts had noticeable enhancements on LAI, with significant correlation at most stages. On average, fertilization increased SPAD, regardless of inoculation, whereas drought decreased this trait. Fertilization had a higher correlation with this trait than did irrigation. Fertilization enhanced NDVI, regardless of inoculation. Drought, on the other hand, decreased NDVI in inoculated plants, whereas it slightly enhanced this trait, on average, in noninoculated plants compared to the fully irrigated regime. However, the half-irrigated regime had the highest NDVI value.

Inoculated plants had, on average, higher LAI values compared to noninoculated counterparts, whereas, interestingly, noninoculated plants had higher SPAD values. Inoculation had a negligible effect on NDVI.

## Figures and Tables

**Table 1:** The effect of different fertilization rates on SPAD at different stages of soybean's (cv. Boglár) life cycle, averaged among 2017, 2018, and 2019 in Debrecen, Hungary.

Inoculation	Stage	0N	35N	105N
Inoculated	V4	38.3 ± 2.6	38.5 ± 2.3	39.6 ± 3.1
	R2	35.9 ± 4.3	37.5 ± 3.7	37.9 ± 3.3
	R4	36.2^b^ ± 3.6	38.1^a^ ± 3.1	39.6^a^ ± 3.3
	R6	39.4^b^ ± 5.1	41.2^ab^ ± 4.2	42.3^a^ ± 3.7
	Average	37.5	38.8	39.9

Noninoculated	V4	38.0^b^ ± 2.9	38.9^ab^ ± 2.5	40.2^a^ ± 2.8
	R2	36.6 ± 4.5	37.8 ± 4.6	38.5 ± 3.6
	R4	36.8^b^ ± 2.7	38.1^b^ ± 2.9	40.5^a^±3.4
	R6	40.3 ± 5.5	40.7 ± 5.0	42.6 ± 4.7
	Average	37.9	38.9	40.4

Different letters indicate significant differences at 0.05 level among fertilization treatments within a certain stage.

**Table 2:** Correlation coefficient of SPAD, NDVI, and LAI traits at different stages with fertilization.

Inoculation	Stage	SPAD	NDVI	LAI
Inoculated	V4	.205^*∗*^	.316^*∗∗*^	.324^*∗∗*^
	R2	.221^*∗*^	.383^*∗∗*^	.383^*∗∗*^
	R4	.386^*∗∗*^	.167	.468^*∗∗*^
	R6	.269^*∗∗*^	−.005	−.006
	Overall	.251^*∗∗*^	.121^*∗*^	.139^*∗∗*^

Noninoculated	V4	.312^*∗∗*^	.117	.269^*∗∗*^
	R2	.181	.017	.280^*∗∗*^
	R4	.456^*∗∗*^	.144	.194^*∗*^
	R6	.192^*∗*^	.003	.069
	Overall	.381^*∗∗*^	.098	.292^*∗∗*^

^*∗*^Correlation is significant at the 0.05 level (2-tailed). ^*∗∗*^ Correlation is significant at the 0.01 level (2-tailed).

**Table 3:** The effect of different irrigation regimes on SPAD at different stages of soybean's (cv. Boglár) life cycle, averaged among 2017, 2018, and 2019 in Debrecen, Hungary.

Inoculation	Stage	Nonirrigated	Half-irrigated	Fully irrigated
Inoculated	V4	39.1 ± 2.6	38.5 ± 3.1	38.7 ± 2.4
	R2	36.8 ± 4.5	37.1 ± 3.7	37.4 ± 3.4
	R4	38.7 ± 3.8	37.5 ± 4.1	37.6 ± 2.9
	R6	38.2^b^ ± 4.5	41.4^a^ ± 2.9	43.3^a^ ± 3.0
	Average	38.2	38.6	39.3

Noninoculated	V4	39.4 ± 3.1	39.3 ± 2.9	38.5 ± 2.5
	R2	37.1 ± 4.1	38.0 ± 4.0	37.9 ± 3.6
	R4	38.7 ± 3.6	38.6 ± 3.7	38.1 ± 2.8
	R6	38.9^b^ ± 5.6	41.1^ab^ ± 3.5	43.6^a^ ± 4.0
	Average	38.5	39.2	39.5

Different letters indicate significant differences at 0.05 level among irrigation regimes within a certain stage.

**Table 4:** Correlation coefficient of SPAD, NDVI, and LAI traits at different stages with irrigation.

Inoculation	Stage	SPAD	NDVI	LAI
Inoculated	V4	−.062	.107	−.009
	R2	.070	−.028	.143
	R4	−.130	.102	.456^*∗∗*^
	R6	.472^*∗∗*^	.240^*∗*^	.194^*∗*^
	Overall	.109^*∗*^	.111^*∗*^	.112^*∗*^

Noninoculated	V4	−.124	−.108	−.146
	R2	.069	−.201^*∗*^	.012
	R4	−.075	.083	.252^*∗∗*^
	R6	.397^*∗∗*^	.126	.134
	Overall	.149	−.019	.132

^*∗*^Correlation is significant at the 0.05 level (2-tailed). ^*∗∗*^ Correlation is significant at the 0.01 level (2-tailed).

**Table 5:** The effect of different fertilization rates on NDVI at different stages of soybean's (cv. Boglár) life cycle, averaged among 2017, 2018, and 2019 in Debrecen, Hungary.

Inoculation	Stage	0N	35N	105N
Inoculated	V4	72.2^b^ ± 5.3	74.0^ab^ ± 5.0	76.1^a^±4.3
	R2	81.9^b^ ± 1.7	82.8^a^ ± 1.6	83.5^a^ ± 1.7
	R4	80.6 ± 3.8	81.4 ± 3.2	82.1 ± 3.8
	R6	79.7 ± 6.4	80.2 ± 7.4	79.6 ± 8.4
	Average	78.6±	79.6	80.3

Noninoculated	V4	73.7 ± 3.9	75.0 ± 5.6	75.0 ± 4.7
	R2	82.2 ± 3.0	82.2 ± 4.3	82.5 ± 5.3
	R4	81.2 ± 3.9	81.7 ± 3.6	82.5 ± 3.5
	R6	79.7 ± 5.7	79.7 ± 6.8	79.7 ± 6.7
	Average	79.2	79.6	79.9

Different letters indicate significant differences at 0.05 level among fertilization treatments within a certain stage.

**Table 6:** The effect of different irrigation regimes on NDVI at different stages of soybean's (cv. Boglár) life cycle, averaged among 2017, 2018, and 2019 in Debrecen, Hungary.

Inoculation	Stage	Nonirrigated	Half-irrigated	Fully irrigated
Inoculated	V4	73.3 ± 5.0	74.4 ± 5.0	74.6 ± 5.3
	R2	82.7 ± 1.6	83.1 ± 1.7	82.5 ± 2.0
	R4	81.0 ± 4.5	81.1 ± 3.0	81.9 ± 3.3
	R6	77.3^b^ ± 5.1	80.6^ab^ ± 5.5	81.6^a^ ± 4.9
	Average	78.6	79.8	80.2

Noninoculated	V4	74.7 ± 3.4	75.6 ± 4.0	73.4 ± 5.3
	R2	82.9 ± 2.1	82.9 ± 2.4	80.9 ± 3.1
	R4	81.4 ± 4.5	81.7 ± 3.5	82.2 ± 3.0
	R6	78.7 ± 6.2	79.7 ± 5.9	80.6 ± 6.8
	Average	79.4	80.0	79.3

Different letters indicate significant differences at 0.05 level among irrigation regimes within a certain stage.

**Table 7:** The effect of different irrigation regimes on LAI at different stages of soybean's (cv. Boglár) life cycle, averaged among 2017, 2018, and 2019 in Debrecen, Hungary.

Inoculation	Stage	Nonirrigated	Half-irrigated	Fully irrigated
Inoculated	V4	1.8 ± 0.3	1.8 ± 0.3	1.9 ± 0.4
	R2	4.5 ± 0.9	4.5 ± 0.8	5.0 ± 0.7
	R4	7.2^c^ ± 0.6	8.2^b^ ± 0.6	8.8^a^ ± 0.8
	R6	5.6 ± 0.7	6.2 ± 1.0	6.3 ± 0.8
	Average	4.8	5.2	5.5

Noninoculated	V4	2.0 ± 0.4	1.8 ± 0.3	1.7 ± 0.4
	R2	4.6 ± 1.0	4.5 ± 0.9	4.7 ± 1.0
	R4	7.2^b^ ± 0.8	7.5^ab^ ± 0.5	8.1^a^ ± 0.9
	R6	5.6 ± 0.6	6.2 ± 1.0	6.1 ± 0.9
	Average	4.9	5.0	5.2

Different letters indicate significant differences at 0.05 level among irrigation regimes within a certain stage.

**Table 8:** The effect of different fertilization rates on LAI at different stages of soybean's (cv. Boglár) life cycle, averaged among 2017, 2018, and 2019 in Debrecen, Hungary.

Inoculation	Stage	0N	35N	105N
Inoculated	V4	1.7^b^ ± 0.3	1.8^b^ ± 0.3	2.2^a^ ± 0.4
	R2	4.0^b^ ± 0.5	4.5^b^ ± 0.7	5.4^a^ ± 0.9
	R4	7.4 ± 0.7	7.7 ± 0.6	9.1 ± 0.6
	R6	6.0 ± 0.7	6.1 ± 0.9	6.1 ± 0.9
	Average	4.8^b^	5.0^b^	5.7^a^

Noninoculated	V4	1.7^b^ ± 0.3	1.8^ab^ ± 0.3	2.1^a^ ± 0.5
	R2	3.9^b^ ± 0.7	4.6^ab^ ± 1.0	5.3^a^ ± 1.2
	R4	7.3 ± 0.7	7.6 ± 0.8	8.0 ± 0.8
	R6	5.8 ± 0.8	6.0 ± 0.7	6.1 ± 1.1
	Average	4.7	5.0	5.4

Different letters indicate significant differences at 0.05 level among fertilization treatments within a certain stage.

**Table 9:** The effect of different fertilization treatments on LAI of soybean (cv. Boglár) under different irrigation regimes, averaged among 2017, 2018, and 2019 in Debrecen, Hungary.

Inoculation	Irrigation regime	0N	35N	105N	Average
Inoculated	Nonirrigated	82.5^b^ ± 12.8	85.1^b^ ± 15.0	88.6^b^ ± 15.2	85.4
	Half-irrigated	86.9^a^ ± 15.3	93.1^a^ ± 16.7	95.4^a^ ± 16.8	91.8
	Fully irrigated	89.8^a^ ± 16.1	91.4^a^ ± 17.4	94.2^a^ ± 16.4	91.8
	Average	86.4	89.9	92.7	89.7

Noninoculated	Nonirrigated	80.7 ± 9.8	84.8 ± 14.9	85.9 ± 13.8	83.8
	Half-irrigated	87.5 ± 11.9	91.6 ± 14.3	93.3 ± 16.3	90.8
	Fully irrigated	86.9 ± 13.5	90.6 ± 14.3	91.6 ± 13.9	89.7
	Average	85.0	89.0	90.2	88.1

In each inoculation treatment, different letters indicate significant differences at 0.05 level among irrigation regimes within a certain fertilization treatment.

## Data Availability

No data were used to support this study.
